# Construction of osteoporosis diagnosis model based on bioinformatics analysis of autophagy-related genes

**DOI:** 10.1097/MD.0000000000044950

**Published:** 2025-10-03

**Authors:** Guoshuai Liu, Hengwu Zhao, Shuo Wang, Shengwu Chen

**Affiliations:** a Graduate Education Base, Third Affiliated Hospital of Jinzhou Medical University, Jinzhou, People’s Republic of China; b Department of Orthopaedics, Third Affiliated Hospital of Jinzhou Medical University, Jinzhou, People’s Republic of China; c Department of Endocrinology, First Affiliated Hospital of Jinzhou Medical University, Jinzhou, People’s Republic of China.

**Keywords:** autophagy, bioinformatics, diagnostic model, GEO database, osteoporosis

## Abstract

Osteoporosis (OP) is a major health issue that poses a substantial challenge to public health worldwide. In recent decades, knowledge about the etiological mechanisms of osteoporosis has emphasized that bone cell homeostasis is tightly regulated by autophagy. However, little is currently known about autophagy-related genes (ATGs) that influence osteoporosis, which compromises our deep understanding of the pathogenesis of osteoporosis and our ability to develop targeted treatment strategies. Here, we screen differentially expressed autophagy-related genes (DE-ATGs) in osteoporosis using GSE56815 dataset and human autophagy database and explore the role of autophagy-related biomarkers in the occurrence and development of OP to provide new ideas for the diagnosis and treatment of OP. The original dataset was downloaded from the gene expression omnibus database and further integrated and analyzed. The differential genes of OP were screened using R software, and the differentially expressed genes of OP were intersected with ATGs obtained from the human autophagy gene pool to obtain DE-ATGs. The differentially expressed autophagy genes were enriched using gene ontology, Kyoto Encyclopedia of Genes and Genomes, Metascape, DisGeNet, and gene set enrichment analysis. Core DE-ATGs were screened using Least Absolute Shrinkage and Selection Operator and cross-validation, and an receiver operating characteristic curve was constructed to explore the diagnostic value of key genes for OP. According to receiver operating characteristic curve analysis, ras-related C3 botulinum toxin substrate 1, epidermal growth factor receptor, and cathepsin D are key genes with high diagnostic value related to autophagy, which can provide a new method for the diagnosis and treatment of OP.

## 1. Introduction

Osteoporosis (OP) is a systemic, degenerative bone disease characterized by a gradual decrease in bone mass and significant degradation of bone mechanical properties, leading to increased bone fragility and a higher risk of fractures.^[1–3]^ As populations age globally, osteoporosis has emerged as a major public health concern, affecting millions and imposing substantial economic, and social burdens.^[4,5]^ Current treatments for osteoporosis, such as bisphosphonates and denosumab, face challenges including suboptimal therapeutic efficacy, notable side effects, and limited ability to enhance osteoblast proliferation or reverse disease progression.^[6,7]^ Consequently, identifying novel diagnostic markers and therapeutic targets is critical to improving clinical outcomes for osteoporosis patients.^[8]^

Autophagy, a dynamic self-digestion process involving the degradation of intracellular proteins and aging organelles, is essential for cell survival and homeostasis under physiological and pathological conditions.^[9–11]^ In the context of bone metabolism, autophagy tightly regulates the function of osteoblasts, osteoclasts, and osteocytes.^[12]^ For instance, autophagy promotes osteoblast survival by mitigating oxidative stress through the mammalian Target Of Rapamycin signaling pathway and supports osteoclast cytoskeletal reorganization and bone resorption via microtubule-associated protein 1A/1Blight chain 3 II (LC3-II)^[13]^ and Beclin-1 regulation.^[14,15]^ Dysregulation of autophagy, such as impaired autophagosome formation, has been linked to increased osteoclast activity and bone loss, particularly in aging and estrogen-deficient states. Studies have shown that autophagy-related genes (ATG), such as autophagy-related gene 5 (ATG5) and autophagy-related gene 7 (ATG7), are pivotal in maintaining bone mass by balancing bone formation and resorption.^[16]^ These findings highlight the potential of ATGs as diagnostic and therapeutic targets for osteoporosis.

Despite these advances, significant gaps remain in translating autophagy research into clinical applications for osteoporosis. Most studies have focused on animal models or cell lines, with limited exploration of ATG expression in human osteoporosis cohorts. Moreover, existing diagnostic approaches for osteoporosis primarily rely on the long-term accumulated professional experience of doctors or imaging techniques like dual-energy x-ray absorptiometry or non-autophagy-related biomarkers, which often lack specificity for early-stage disease.^[17]^ The integration of ATGs into diagnostic models remains underexplored, particularly in the context of large-scale human transcriptomic data. This gap hinders the development of precise, noninvasive diagnostic tools and targeted therapies that address the autophagic dysregulation underlying osteoporosis progression.

In recent years, bioinformatics has revolutionized our ability to probe disease pathobiology at the genetic level.^[18]^ Public databases, such as the Gene Expression Omnibus (GEO),^[19]^ provide extensive transcriptomic data, enabling cost-effective and in-depth analyses of molecular mechanisms. While researchers have identified differentially expressed genes (DEGs) associated with osteoporosis using these resources, few studies have specifically investigated the intersection of osteoporosis and ATGs. This study aims to address these gaps by leveraging bioinformatics to identify and validate DE-ATGs in osteoporosis using human transcriptomic data from the GEO database. Specifically, we seek to construct a diagnostic model based on DE-ATGs to predict osteoporosis risk with high accuracy and explore their biological roles in bone metabolism. By integrating gene expression profiling with functional enrichment and immune cell correlation analyses, we aim to provide novel biomarkers for early diagnosis and potential therapeutic targets for autophagy-modulating treatments in osteoporosis.

In this study, we conducted an analysis of the GEO series56815 (GSE56815)^[20]^ and GEO series2208 (GSE2208)^[21]^ gene chip datasets. Utilizing R for screening and in-depth mining, we identified key DEGs. By integrating with the autophagy gene database, we established a diagnostic model based on DE-ATGs in OP. This model has enhanced the accuracy and convenience of OP diagnosis, providing more biomarkers for clinical work and research into the mechanisms of OP.

## 2. Materials and methods

### 2.1. Data sources

In this study, the GSE56815 dataset was downloaded from the GEO database as an experimental group and the platform annotation file GPL96 was downloaded from the database website for probe ID and gene name conversion. The dataset came from 80 Caucasian female blood samples collected by the Tulane University School of Public Health in Los Angeles, USA, including 40 high bone density female blood monocyte samples and 40 low bone density female blood monocyte samples. The GSE2208 dataset was downloaded from the website as the model diagnostic efficacy verification group, which included 10 blood monocyte samples from women with high bone mineral density (BMD) women and 9 blood monocyte samples from women with low BMD women.

### 2.2. Data processing

The original and serial matrix files of the microarray dataset for the training dataset GSE56815 and the validation dataset GSE2208 were downloaded. The random effects meta-analysis ^[22]^ in the R package is used to extract and standardize the probe expression matrix from the original data, and then transform it into a gene expression matrix through the platform annotation file. Multiple probes corresponding to the same gene were then averaged. After obtaining the transformed gene expression matrix using the Perl script, the normalized gene expression matrix was analyzed using Limma R package.^[23]^

### 2.3. Screening and analysis of differential genes related to autophagy

To identify DEGs between OP and healthy individuals, the Limma package was used to screen DEGs according to Adj. *P* value ≤ .05 and |log FC| ≥ 0.1. At the same time, ATGs related to human diseases were obtained from the human autophagy database (http://www.autophagy.lu/), and then ATGs and DEGs were intersected by using the VennDiagram package^[24]^ in the R to obtain DE-ATGs. DE-ATGs are visualized by drawing heat maps and volcanic maps through heatmap package^[25]^ and ggplot2 packages,^[26]^ respectively.

### 2.4. Functional enrichment analysis

Gene set enrichment analysis (GSEA) was used to analyze DEGs between OP and healthy individuals using the FGSEA package^[27]^ to visualize the biological pathways that differ between OP and healthy individuals. DE-ATGs were analyzed and visualized using Gene Ontology (GO) and Kyoto Encyclopedia of Genes and Genomes (KEGG) by using the KOBAS database.^[28]^ The screening condition was Adj. *P* value ≤ .05, and the biological pathways of the DEGs related to autophagy were further studied. DisGeNet^[29]^ enrichment analysis of DE-ATGs was performed using the Metascape database^[30]^ to determine which diseases were most closely related to them.

### 2.5. Establishment and evaluation of risk scoring diagnosis model based on DE-ATGs

Using glmnet package,^[31]^ DE-ATGs are analyzed with minimum absolute contraction and Least Absolute Shrinkage and Selection Operator (LASSO) regression operator (LASSO)^[32]^ to constrain parameters, prevent over-fitting, and reduce bias. The OP characteristic genes identified by LASSO regression were analyzed using multivariate logistic analysis. Statistical significance was defined as *P* ≤ .05, and the risk score was calculated by multiplying the expression level of AT-DEG (α) by the corresponding coefficient (β). The results were evaluated and analyzed using a line graph model, and the incidence of DE-ATGs in OP was predicted. Finally, the pROC package^[33]^ was used to evaluate the accuracy of the prediction model and calculate the area under the receiver operating characteristic curve (AUC, area under the curve).

### 2.6. Construction and verification of nearline diagram

Using RMS^[34]^ to construct a column chart, the meaningful parameters obtained from logistic regression analysis are summarized, and multiple predictors are integrated to evaluate the occurrence probability of OP. Harrell consistency index (*C*-index)^[35]^ is used to calculate the probability that the predicted results are consistent with the actual observation results, and the calibration curves are drawn by Hmisc (https://www.r-project.org/) and RMS to visualize the analysis results and evaluate the prediction ability of the risk scoring model based on DE-ATGs.

## 3. Results

### 3.1. Screening of DEGs and functional implications

**Figure 1.**
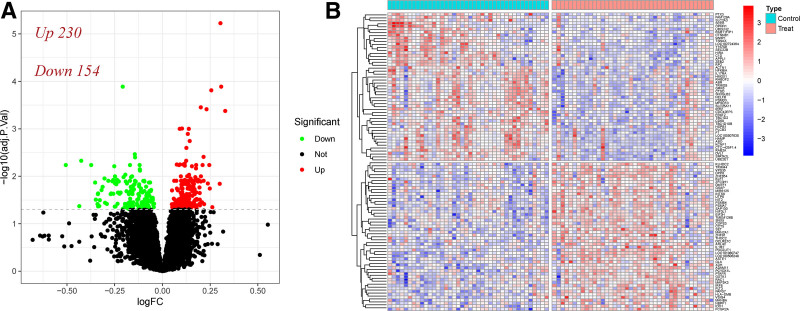
Screening and identification of differential genes. (A) The volcano map shows up-regulated genes, up-regulated genes represented by red scatter points, down-regulated genes represented by green scatter points, and undifferentiated genes represented by black scatter points. (B) The heatmap shows the top 50 genes with the most significant changes in up-regulated and down-regulated genes, which were screened according to the absolute value of log_2_ FC.

**Figure 2.**
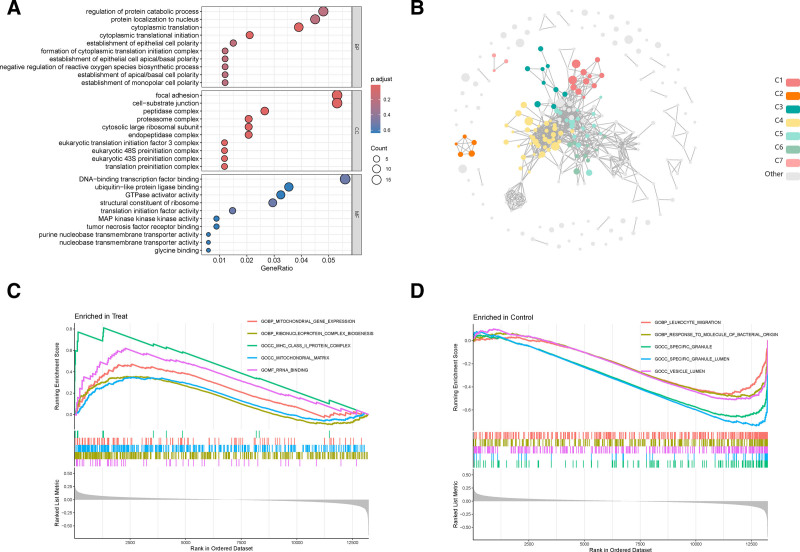
The function analysis of DEGs. (A) The GO enrichment of all DEGs. (B) The KEGG enrichment and cluster enrichment of all DEGs using KOBAS database. (C) The GSEA analysis of treat condition. (D) The GSEA analysis of control condition. DEGs = differentially expressed genes, GO = gene ontology, GSEA = gene set enrichment analysis, KEGG = Kyoto Encyclopedia of Genes and Genomes.

By analyzing the data contained in the training dataset GSE56815, A total of 384 DEGs were screened based on the threshold (see Section 2). Through volcanic and heatmap, it can be seen that there are significant differences in the expression levels of DEGs between patients with OP and healthy individuals. Among them, 230 genes were upregulated and 154 genes were downregulated (Fig. 1A). At the same time, we found key genes solute carrier family 25 (Mitochondrial Carrier; Oxoglutarate Carrier), member 11, solute carrier family 25 Member 11, significantly down-regulated in the treatment group. Down-regulation of solute carrier family 25 member 11 could potentially affect mitochondrial function and the citric acid cycle, which might have implications for cellular energy production and other metabolic processes^[36]^ (Fig. 1B). To explore the function of differential genes, we performed GO, KEGG enrichment and GSEA analyses on differential genes. GO function enrichment analysis of DEGs included cellular component, biological process (BP), and molecular function, which mainly included regulation of protein catabolism, nuclear localization of proteins, adhesion plaques, cell-matrix junctions, DNA-binding transcription factor binding, and ubiquitin-like protein ligase binding (Fig. 2A). The KEGG results showed that the differential genes were divided into 7 classes, which were grouped into Pathways in cancer-related terms, and other signaling pathways were also enriched (Figs. 2B, S2, Supplemental Digital Content, https://links.lww.com/MD/Q199). GSEA enrichment analysis of 384 DEGs between OP and healthy individuals identified 42 potential biological pathways (Fig. 2C, D). Leukocyte migration, reaction of bacteria-derived molecules, special particles, special particle lumen, and vesicle cavity pathways were the most active mitochondrial gene expression pathways in the normal group, and ribonucleoprotein complex biogenesis, major histocompatibility complex II protein complex, mitochondrial matrix, and rRNA binding pathways were the most active biological pathways in the OP group.

### 3.2. Identification of DE-ATGs and function analysis

**Figure 3.**
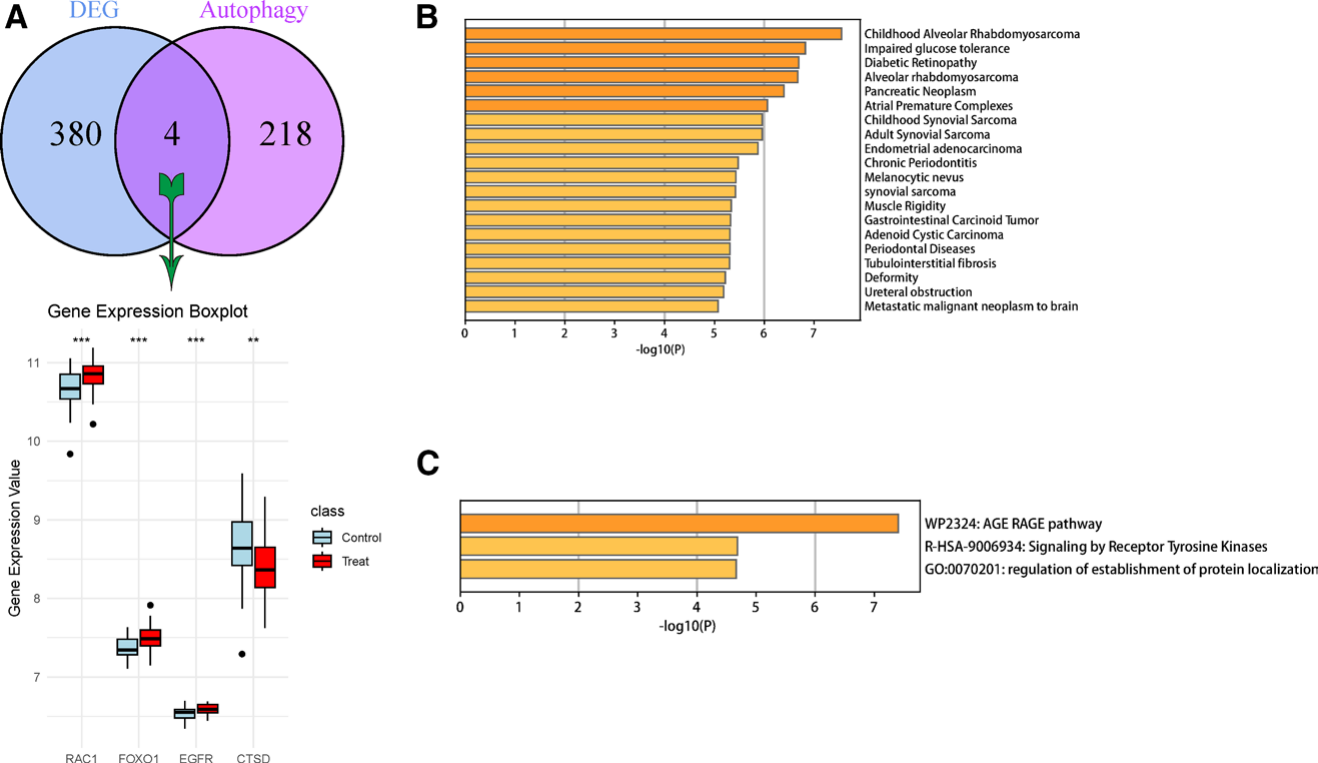
Identification of DE-ATGs and function analysis. (A) At the top of the figure is the overlap analysis of DEG and ATG, while at the bottom is the gene expression box visualization of overlapping genes. Significance tests were conducted for the experimental group and the control group using *T*-student test, with *** representing *P* ≤ .001 and ** representing *P* ≤ .01. (B) The enrichment pathway using DisGeNET. (C) The GO enrichment pathway using metascape. DE-ATGs = differentially expressed autophagy-related genes, DEGs = differentially expressed genes, GO = gene ontology.

By comparing these DEGs with 222 ATGs collected from human autophagy database, we found that 4 DE-ATGs were intersecting, namely, ras-related C3 botulinum toxin substrate 1 (RAC1), forkhead box O1 (FOXO1), epidermal growth factor receptor (EGFR), and cathepsin D (CTSD). According to the boxplot, CTSD was downregulated in the experimental group and upregulated in the control group, while RAC1, FOXO1, and EGFR were upregulated in the experimental group and downregulated in the control group (Fig. 3). Four DE-ATGs were identified and subjected to comprehensive analysis using the R software. The GO functional enrichment analysis of these DE-ATGs was conducted across 3 primary domains: cellular component, BP, and molecular function. This analysis encompassed a spectrum of biological pathways and functions, such as the Wnt signaling pathway, Wnt-mediated cell-cell signaling transduction, the insulin receptor signaling pathway, and the regulation of reactive oxygen species metabolism. Additionally, it included cellular structures and functions like the wrinkle membrane and pigment granules. The findings provide a nuanced understanding of the roles these genes play in various biological processes and pathways (Fig. S1, Supplemental Digital Content, https://links.lww.com/MD/Q199). To further explore the function of these genes, we performed functional analyses using metascape and DisGeNET, the result showed that DE-ATGs are closely related to advanced glycosylation end-product-receptor for advanced glycation end-products signaling pathway (Fig. 3C), alveolar rhabdomyosarcoma, atrial premature beat complex, pancreatic tumor, alveolar rhabdomyosarcoma, diabetic retinopathy, and diseases other than osteoporosis in children (Fig. 3B).

### 3.3. Immune cell distribution and correlation with DE-ATGs in nonneoplastic diseases

**Figure 4.**
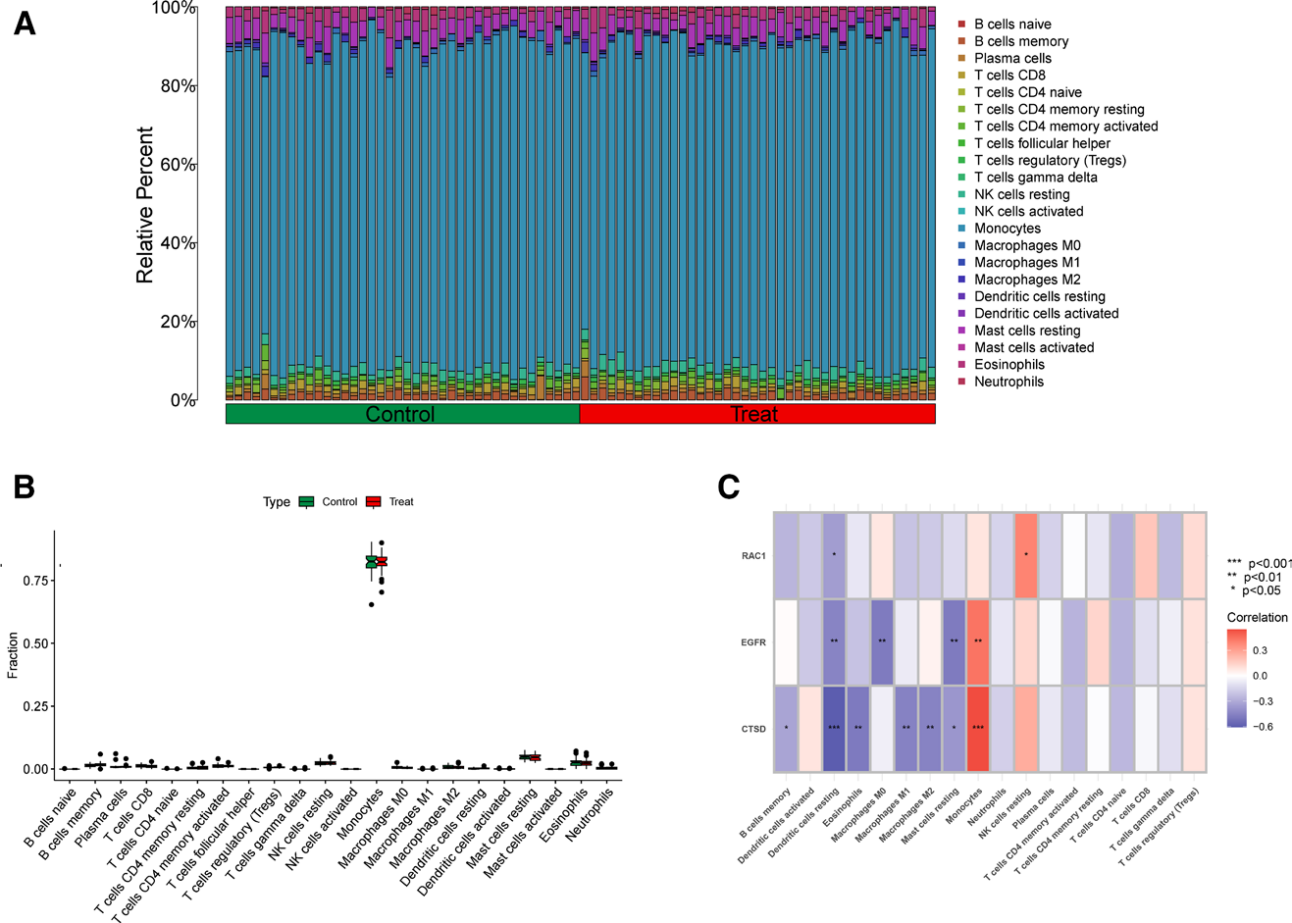
Immune cell distribution and correlation with DE-ATGs in osteoporosis. (A) Bar plot showing the relative proportions of immune cell types in the control and experimental (osteoporosis) groups, as analyzed by CIBERSORT. Monocytes are the predominant cell type in both groups. (B) Box plot comparing the proportion of monocytes between the control and experimental groups, indicating no significant difference. (C) Correlation heatmap illustrating the relationships between key differentially expressed autophagy-related genes (DE-ATGs: RAC1, EGFR, CTSD) and immune cell types. Positive correlations are shown in red, and negative correlations are shown in blue. Significant correlations include CTSD with monocytes (positive), EGFR with resting dendritic cells (negative), and RAC1 with resting NK cells (positive). DE-ATGs = differentially expressed autophagy-related genes, CIBERSORT = classification Identification by estimation of relative subpopulations, CTSD = cathepsin D, EGFR = epidermal growth factor receptor, NK cells = natural killer cells, RAC1 = ras-related C3 botulinum toxin substrate 1.

In nonneoplastic diseases, the infiltration and functional status of immune cells are critical to the onset, progression, and treatment response of the disease.^[37]^ Therefore, in order to evaluate and understand the distribution, abundance, and functional status of immune cells in tissues. We used classification identification by estimation of relative subpopulations for analysis, and the results showed that monocytes accounted for the highest proportion in both the control group and the experimental group, and were far more than other immune cell types (Fig. 4A). However, there was no difference in the proportion of monocytes between the control group and the experimental group (Fig. 4B). Further, in order to examine the relationship between 3 key DE-ATGs and immune cells, we conducted a correlation analysis of immune cells, and the results showed that CTSD was positively correlated with monocytes, negatively correlated with resting dendritic cells, EGFR monocytes, and negatively correlated with resting dendritic cells, macrophages M0, and resting mast cells. RAC1 was positively correlated with resting natural killer cells and negatively correlated with resting dendritic cells (Fig. 4C).

### 3.4. Establishment, evaluation and verification of DE-ATGs related diagnostic model

**Figure 5.**
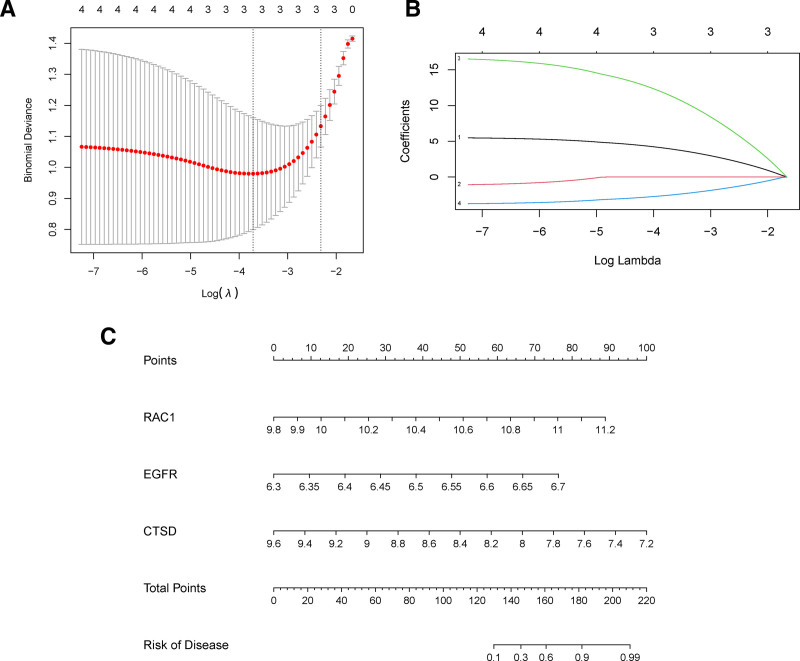
Establishment of a multipredictor nomogram and DE-ATG selection using the LASSO and logistic regression models. (A) Cross-validation to select the most suitable tuning parameter lambda (λ); a λ value of 0.01685 with log(λ) being −3.793 was chosen as optimal. (B) The coefficients in the LASSO regression model for key DE-ATGs. (C) Predictive nomogram involving age, sex, and the expression profile of 3 DE-ATGs based on selected features. DE-ATGs = differentially expressed autophagy-related genes, LASSO = Least Absolute Shrinkage and Selection Operator.


To improve the accuracy, efficiency and accessibility of disease diagnosis, it is necessary to build a diagnostic model. Therefore, to construct a diagnostic model for osteoporosis, LASSO regression analysis was used to minimize the bias of the diagnostic model, ensure the best fitting error, make the parameters as simple as possible, reduce the probability of overfitting, and select the most appropriate tuning parameter λ. Among the 4 candidate genes in the experimental group, 3 key DE-ATGs related to diagnosis were identified: RAC1, EGFR, and CTSD (Fig. 5A). These 3 key genes were used to construct prediction models. The model was constructed using the following formula:

$$\begin{array}{l} \begin{array}{lll} & Risk\;Score=RAC1\cdot 5.36372611284279\; & +EGFR\cdot 16.1160221096208+CTSD\cdot \left(-3.51924031934624\right)\; \end{array} \end{array}$$

Subsequently, a diagnostic model line diagram was established and the results were visualized and applied to clinical practice (Fig. 5B). According to the expression levels of the 3 key DE-ATGs in the human body, doctors or patients can locate them on the corresponding scale of the nematic diagram and calculate their corresponding scores. The cumulative expression score of each gene was calculated as the total score. The risk score projected downward from the total score could be used to evaluate the risk probability of individuals with OP (Fig. 5C).

**Figure 6.**
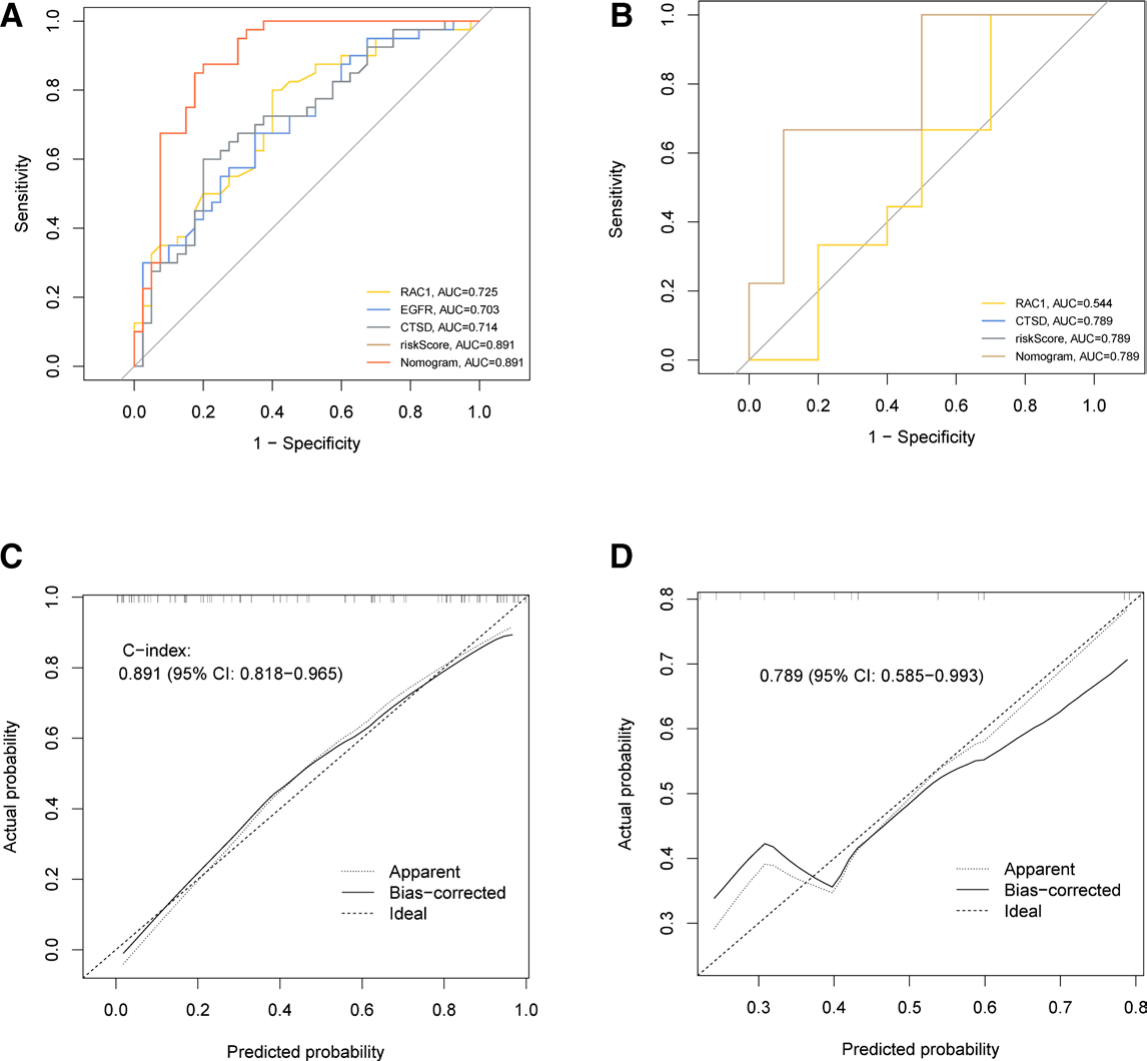
Model discrimination and calibration curve analysis. (A) ROC curve for the prognostic model of OP based on GSE56815. (B) ROC curve of the OP prognostic model based on GSE2208. (C) Calibration curve of the OP nomogram prediction in the GSE56815 set. (D) Calibration curve of the OP nomogram prediction in the GSE2208 set. GSE = gene expression omnibus series, OP = osteoporosis, ROC = receiver operating characteristic.

AUC was used to verify the accuracy of the DE-ATG-related diagnostic model. The area under the AUC curve of the experimental group was 0.891 (Fig. 6A), indicating high accuracy of the model. The AUC of the validation group was 0.789 (Fig. 6B), confirming that the DE-ATGs diagnostic model had high prediction accuracy. *C*-index was used to determine the alignment diagram. The *C*-index of the experimental group was 0.891 (Fig. 6C) and that of the validation group was 0.789 (Fig. 6D), indicating that the prediction ability of the model is worthy of recognition. Based on the area map under the AUC curve of the experimental and verification groups, it was suggested that the RAC1 and CTSD genes had the highest diagnostic accuracy.

## 4. Discussion

Osteoporosis (OP) is a debilitating condition characterized by reduced bone strength and increased fracture risk, often diagnosed late due to the lack of sensitive early biomarkers. Our study uses bioinformatics to identify DE-ATGs in OP, highlighting RAC1, EGFR, and CTSD as key diagnostic markers. By developing a diagnostic model with high predictive accuracy (AUC = 0.891 in GSE56815, 0.789 in GSE2208), we provide a novel, noninvasive approach for early detection and potential therapeutic targeting of autophagy dysregulation in OP. This discussion explores the biological roles of these genes, the clinical implications of our model, comparisons with existing research, and future research directions.

### 4.1. Biological roles of RAC1, EGFR, and CTSD in osteoporosis

Autophagy plays a critical role in bone homeostasis by regulating osteoblast survival, osteoclast differentiation, and osteocyte maintenance. Our findings identify RAC1, a Rho family GTPase, as a key regulator of osteoclast function.^[38]^ RAC1 mediates cytoskeletal reorganization and bone resorption through the phosphatidylinositol 3kinase/protein kinase B (PI3K/Akt) pathway and supports autophagosome formation via LC3-II recruitment.^[39]^ Its upregulation in OP samples suggests increased osteoclast activity, consistent with studies showing that RAC1 inhibition enhances bisphosphonate efficacy in reducing bone resorption.^[40]^ Similarly, EGFR, a tyrosine kinase receptor, influences bone metabolism via PI3K/Akt and mitogen-activated protein kinase pathways, modulating autophagy and osteoblast proliferation.^[41]^ Its overexpression in OP may reflect compensatory mechanisms to counteract bone loss, though dysregulated EGFR signaling could exacerbate autophagic imbalance.^[42]^ In contrast, CTSD, encoding the lysosomal protease Cathepsin D, was downregulated in OP samples, potentially impairing autophagic flux and lysosomal degradation in bone cells.^[43]^ This aligns with evidence linking reduced CTSD activity to increased osteoclast survival and bone loss.^[44,45]^ Although FOXO1, another DE-ATG, was identified, it was excluded from our diagnostic model due to lower expression variability or collinearity with RAC1 and EGFR. However, FOXO1’s role in osteoblast differentiation via oxidative stress regulation merits further investigation.^[46]^

### 4.2. Clinical implications of the diagnostic model

Our diagnostic model, incorporating RAC1, EGFR, and CTSD, offers significant clinical potential. The risk score is calculated as:

Risk Score = RAC1 × 5.36372611284279 + EGFR × 16.1160221096208 + CTSD × (−3.51924031934624). This model, based on blood monocyte gene expression, provides a noninvasive alternative to dual-energy x-ray absorptiometry, which is costly and less sensitive to early-stage OP. The nomogram, integrating gene expression with age and sex, enables personalized risk assessment, facilitating early intervention in high-risk individuals, such as postmenopausal women. Compared to existing biomarkers like serum C-terminal telopeptide of type I collagen or Procollagen type I N-terminal propeptide, our model’s AUC (0.789–0.891) indicates superior predictive accuracy for distinguishing low BMD. The model’s focus on DE-ATGs underscores the role of autophagy dysregulation in OP, consistent with animal models showing that reduced autophagy in osteoblasts and osteocytes correlates with bone loss during aging.^[47]^ Systemic rapamycin administration in aged rats activated autophagy and alleviated age-related OP, supporting the therapeutic potential of targeting autophagy.^[48,49]^

### 4.3. Limitations and future directions

Despite its strengths, our study has limitations. The GEO database, while comprehensive, lacks complete gene coverage, potentially missing other relevant DE-ATGs. Validation in diverse populations, including men and younger individuals, is needed to ensure generalizability. Standardized assays for measuring gene expression in clinical settings are also required to translate our model into practice. Future studies should use animal or cellular models to validate the roles of RAC1, EGFR, and CTSD in OP progression and explore therapeutic interventions, such as autophagy activators (e.g., rapamycin) or RAC1 inhibitors, to mitigate bone loss. Additionally, the role of FOXO1 and other DE-ATGs in OP warrants further exploration, particularly their interactions with oxidative stress and metabolic pathway.^[50]^

In conclusion, our diagnostic model, leveraging RAC1, EGFR, and CTSD, provides a high-sensitivity, noninvasive tool for early OP detection. By targeting autophagy dysregulation, this model offers a promising avenue for personalized risk assessment and therapeutic development, addressing a critical gap in OP management.

## Acknowledgments

We would like to thank Dr Chang Jianye of University College Dublin for his valuable suggestions on this paper.

## Author contributions

**Conceptualization:** Shengwu Chen.

**Data curation:** Guoshuai Liu, Hengwu Zhao, Shengwu Chen.

**Formal analysis:** Guoshuai Liu, Shuo Wang.

**Funding acquisition:** Shengwu Chen.

**Investigation:** Guoshuai Liu, Shuo Wang.

**Methodology:** Hengwu Zhao, Shengwu Chen.

**Project administration:** Shuo Wang, Shengwu Chen.

**Software:** Shuo Wang.

**Validation:** Guoshuai Liu, Hengwu Zhao.

**Writing – original draft:** Guoshuai Liu, Hengwu Zhao.

**Writing – review & editing:** Guoshuai Liu, Shengwu Chen.

## Supplementary Material
