# Supplementary material for: Construction of osteoporosis diagnosis model based on bioinformatics analysis of autophagy-related genes
Source: Medicine (Baltimore). 2025 Oct 3;104(40):e44950. doi: 10.1097/MD.0000000000044950 (PMC12499686; doi:10.1097/MD.0000000000044950)

**Supplemental Figure 1.:** The Gene Ontology (GO) functional enrichment analysis of four differentially expressed autophagy-related genes (DE-ATGs).


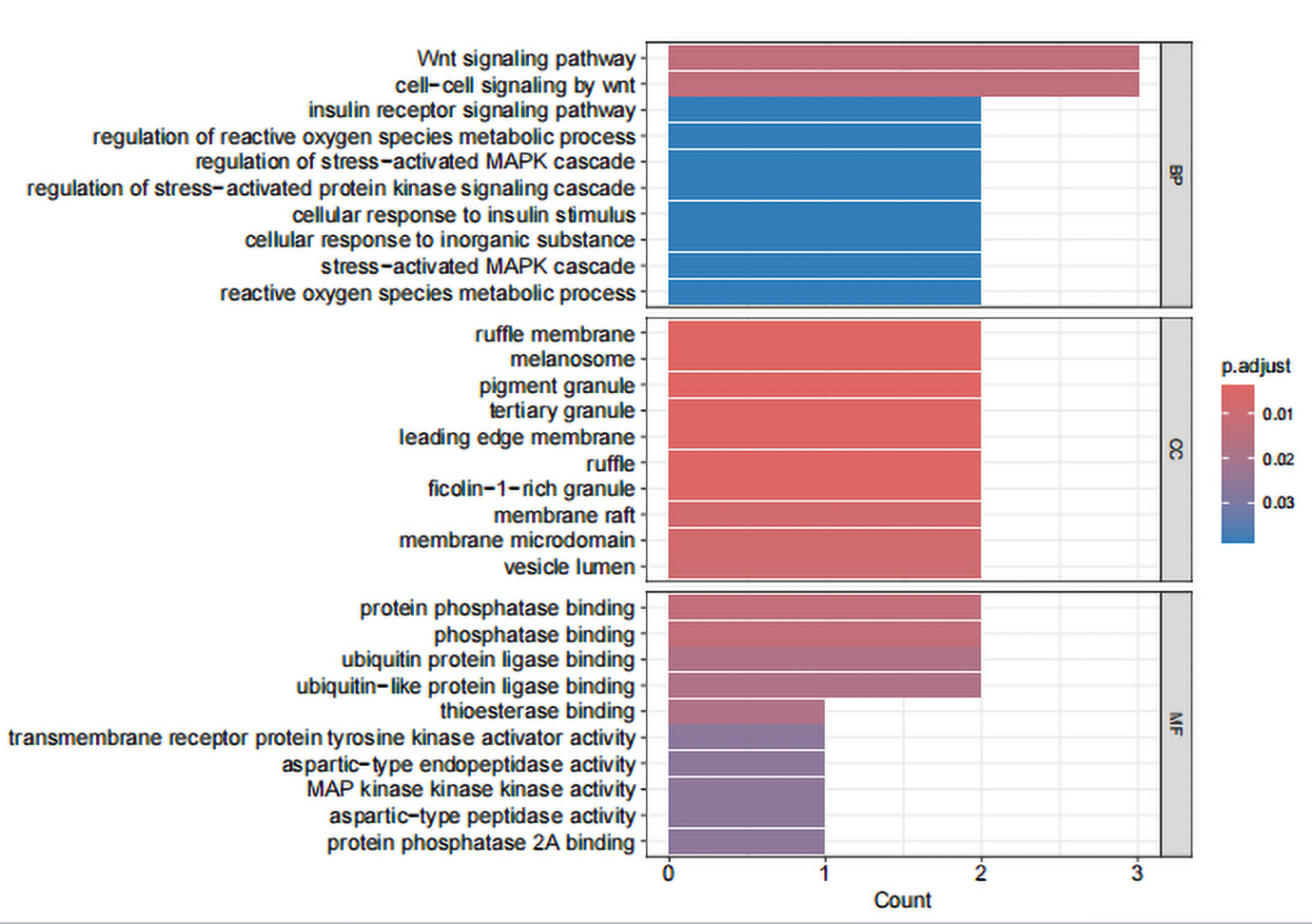


**Supplemental Figure 2:** The KEGG enrichment and cluster enrichment of all DEGs using KOBAS database.


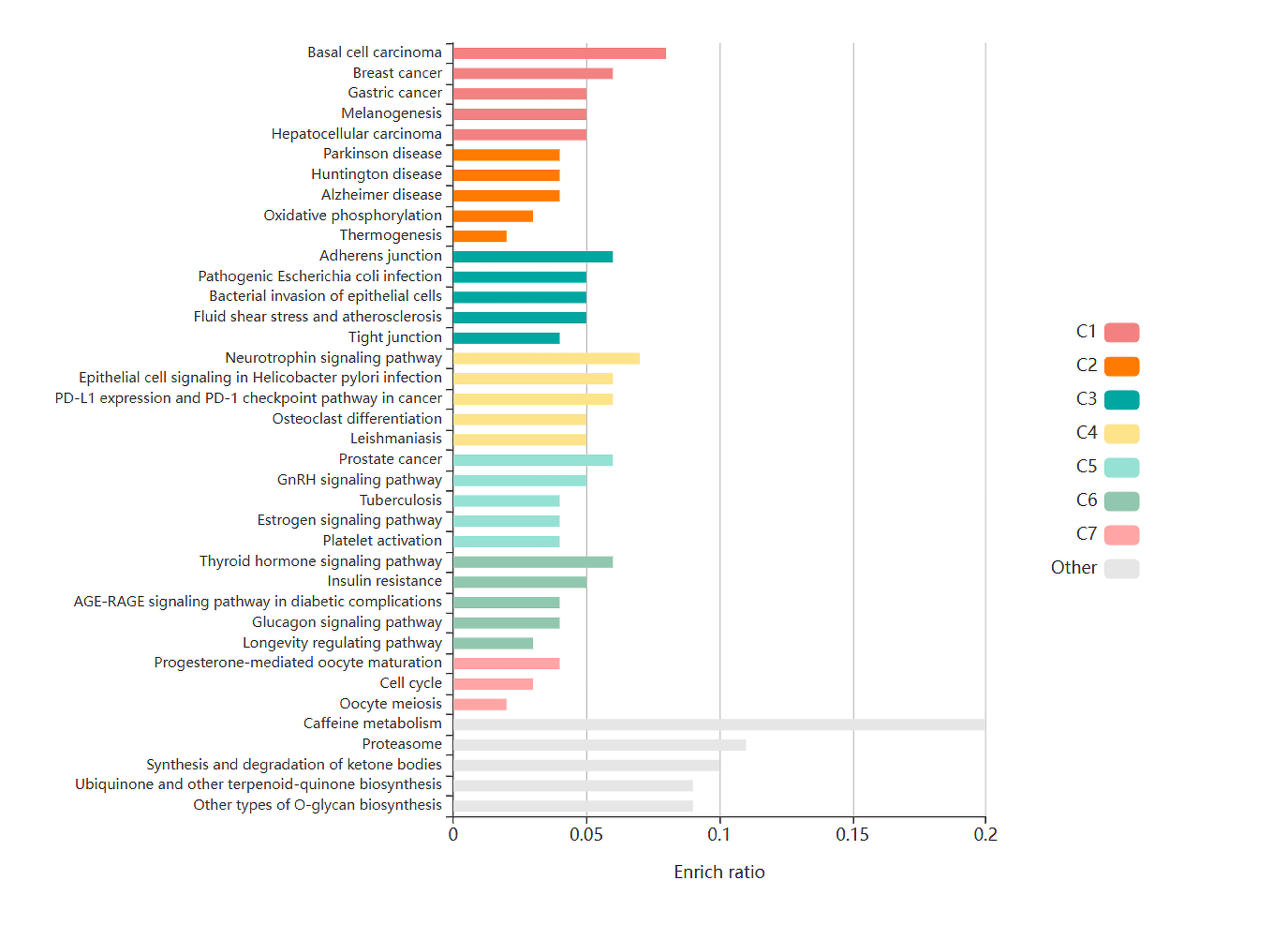

Supplement: Supplementary file 1 [file medi-104-e44950-s001.docx]
